# Challenges in Diagnosing and Differentiating IgG4-Related Disease From Sjögren’s Disease: A Case Report and Literature Review

**DOI:** 10.7759/cureus.83090

**Published:** 2025-04-27

**Authors:** Dosbai Saparov, Aleksandr Markov, Sarang Choi, Shakirat Gold-Olufadi, Henry Becerra, Philipp Barakat, Neharika Shrestha, Yevheniia Andriushchenko, Avezbakiyev Boris, Sima Terebelo

**Affiliations:** 1 Internal Medicine, Brookdale University Hospital Medical Center, New York, USA; 2 Hematology and Oncology, Brookdale University Hospital Medical Center, New York, USA; 3 Rheumatology, Brookdale University Hospital Medical Center, New York, USA

**Keywords:** igg4 disease, igg4 related diseases, igg4 related sialadenitis, immunoglobulin g-4 (igg4) related systemic disease, malignancy, mgus, monoclonal gammopathy of undetermined significance (mgus), risk of malignancy, sjogren, sjogren and sicca

## Abstract

IgG4-related disease (IgG4-RD) is a multi-organ fibroinflammatory condition often misdiagnosed due to its clinical similarities with other rheumatologic diseases such as Sjögren's syndrome (SS) and malignancy. This case highlights a 76-year-old woman with a complex medical history who presented with symptoms initially suggestive of SS but was ultimately diagnosed with IgG4-RD.

The patient in our case report presented with bilateral, large, nontender submandibular lymphadenopathy. Initial serological tests were negative for SSA and SSB markers but revealed elevated immunoglobulin G levels of 2811 mg/dL. Imaging showed enlarged submandibular glands and lymphadenopathy. A core biopsy revealed a dense nodular lymphocytic infiltrate with variable parenchymal fibrosis and salivary gland acinar atrophy. The specimen was sent for further immunohistochemistry (IHC) testing as extended lab workup showed IgG4 levels of 843 mg/dL, raising concern for underlying IgG4-RD. IHC showed an increased number of IgG4 plasma cells, up to 50% in some areas, confirming the diagnosis of IgG4-RD. Treatment with prednisone led to rapid symptomatic improvement.

Differentiating IgG4-RD from SS is challenging due to overlapping clinical and histological features. Elevated serum IgG4 levels (>135 mg/dL) are suggestive but not definitive for IgG4-RD; biopsy remains the gold standard for diagnosis. This case underscores the importance of considering IgG4-RD in patients presenting with SS-like symptoms, especially in the absence of anti-Ro/SSA and anti-La/SSB antibodies, and of initiating an extensive workup to fully delineate the extent of the disease in order to initiate timely treatment and prevent organ damage.

This case report emphasizes the need for thorough evaluation in patients with SS-like symptoms to differentiate IgG4-RD, ensuring accurate diagnosis and effective treatment. Prompt recognition and management of IgG4-RD can improve patient outcomes and prevent long-term morbidity.

## Introduction

IgG4-related disease (IgG4-RD) is an immune-mediated multi-organ fibroinflammatory condition [1]. There are four common phenotypes of IgG4-RD: pancreatic-hepato-biliary disease, retroperitoneal fibrosis and/or aortitis, head and neck-limited disease, and Mikulicz syndrome with systemic involvement [2]. Pancreatic involvement, sclerosing cholangitis, and retroperitoneal fibrosis are more common in males, while Mikulicz syndrome and thyroid involvement are likely to be seen in females [3]. The incidence of IgG4-RD in the USA in 2023 is 1.39 per 100,000 people annually [4]. High levels of IgG4 are the only positive serologic marker of this disease, albeit with moderate sensitivity and specificity of 87.2% and 82.6%, respectively, using the pooled data analysis by Xu et al. Notably, serum measurements of elevated IgG4 are found in approximately 70% of patients with IgG4-RD [5,6]. As IgG4-RD is a multi-organ disease with different symptoms, it often can resemble malignancy or other autoimmune diseases such as Sjögren's syndrome (SS) and vasculitis [7]. Early recognition of IgG4-RD is crucial, as it can lead to serious organ damage if undiagnosed until advanced stages [8]. A favorable response to corticosteroids is pathognomonic for this disease, and failure to respond to corticosteroids excludes the diagnosis according to the American College of Rheumatology diagnostic criteria for IgG4-RD [7]. Glucocorticoid monotherapy is the standard first-line agent for remission induction; however, patients with multisystem or extensive disease are frequently treated with other immunosuppressive agents, such as rituximab, as steroid-sparing therapy to aid in remission induction and maintenance therapy [9,10].

## Case presentation

We present a 76-year-old woman with a past medical history of hypertension, glaucoma, chronic hepatitis C (treated previously with ledipasvir and sofosbuvir, with subsequent undetectable hepatitis C virus (HCV) RNA), chronic lower back pain without sciatica, and prediabetic mellitus.

The patient was in good health until October 2021, when she was incidentally noted to have bilateral, large, firm, nontender submandibular glands and was sent to hematology oncology for evaluation. The patient also complained of dry mouth. There was no fever, chills, weight loss, loss of appetite, or night sweats. There was no family history of cancer. On physical examination, there was bilateral submandibular gland swelling, 1x1 cm on the right side and 2x2 cm on the left side. The masses were firm, partly mobile, and moved with swallowing.

A CT scan of the neck showed mildly enlarged bilateral submandibular glands, a mildly enlarged 1.1 cm x 0.7 cm right level 1B lymph node, and multiple scattered subcentimeter upper cervical lymph nodes. A CT scan of the chest, abdomen, and pelvis (CT CAP) was performed and did not show any evidence of malignancy or metastasis. There was no lymphadenopathy, and visualized abdominal and pelvic structures were unremarkable.

An ultrasound-guided fine-needle aspiration (FNA) biopsy of the left submandibular gland was performed. The cytology report showed a heterogeneous lymphoid population with predominantly small lymphoid cells and a few scattered intermediate lymphocytes, along with rare lymphohistiocytic infiltrates. Lymphocytes showed mixed staining with CD20 and CD2 immunostains. CD5 showed rare positive lymphocytes. CD10 and CKA1/AE3 were negative. These findings suggested a reactive lymph node with a possibility of low-grade lymphoproliferative disorder.

The patient subsequently returned for care a year later in 2022, reporting that the masses had resolved but then recurred. This time, she was also experiencing weight loss of 12 pounds and continued experiencing dry mouth, dry eyes, cough, and dyspnea on exertion.

**Figure 1:**
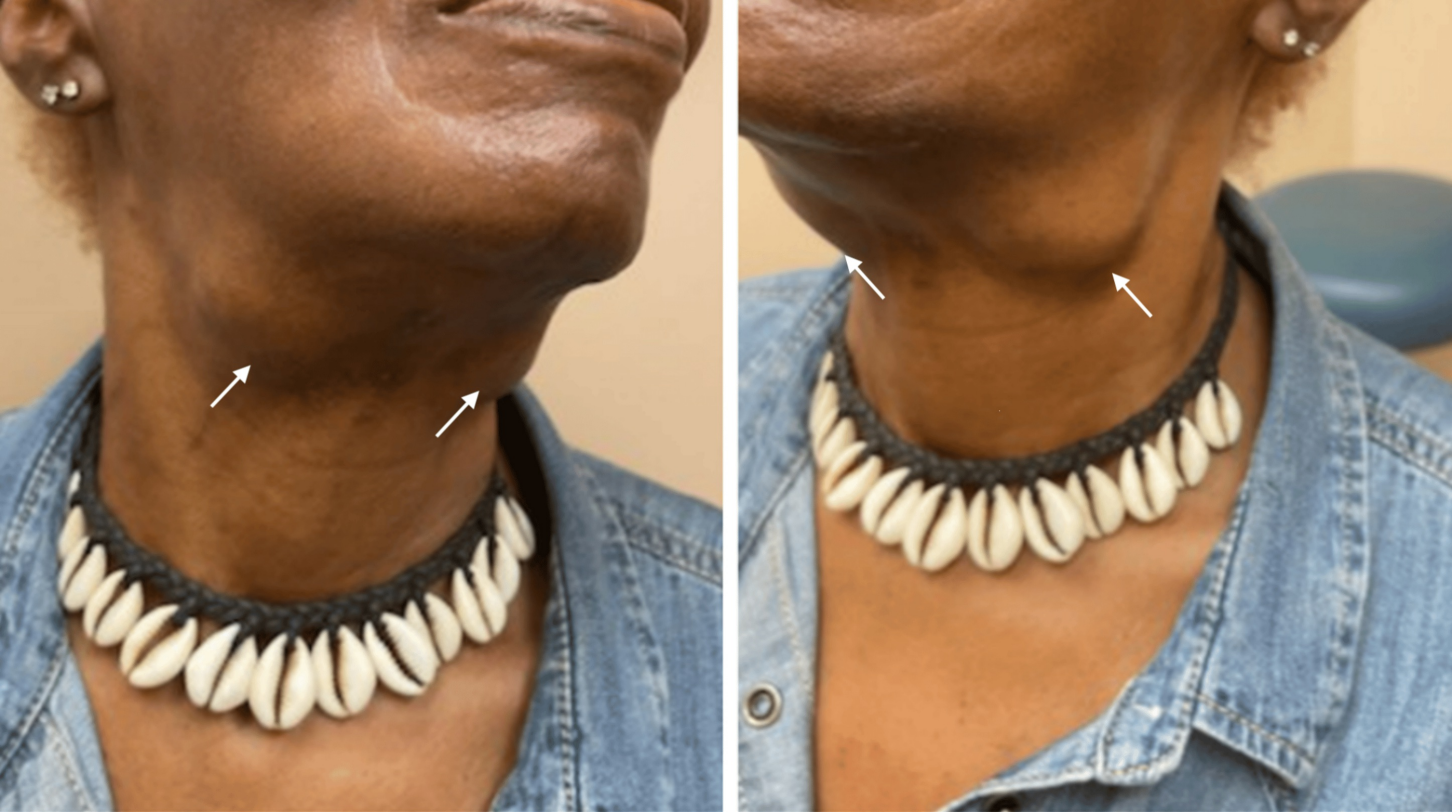
Bilateral enlarged nontender submandibular glands below the mandible (arrows)

The patient was sent to rheumatology for further evaluation of suspected SS. The physical exam was notable for large, firm, nontender submandibular glands bilaterally (Figure 1). There was no joint pain, swelling, synovitis, or effusions. Autoimmune workup showed reactive rheumatoid factor (RF) and anti-cyclic citrullinated protein (CCP) antibodies at high titers (Table 1); however, the patient did not exhibit any clinical signs of inflammatory arthritis. CT of the neck was repeated and showed slightly prominent bilateral submandibular glands with no underlying nodule or calcification. There was no cervical lymphadenopathy.

**Table 1:** Autoimmune panel and inflammatory markers Bolded values indicate abnormal results

^Serologic marker^	^Result (Reference range)^
^Rheumatoid factor (RF)^	^113.6 IU/mL^ ^ (0-14 IU/mL)^
^Anti-cyclic citrullinated peptide antibodies (CCP)^	^>250 U/mL^ ^ (0-20 U/mL)^
^Anti-cardiolipin antibodies (aCL) IgG^	^23 GPL^ ^(0-15 GPL)^
^Anti-cardiolipin antibodies (aCL) IgM^	^37 MPL^ ^(0-12 MPL)^
^Antinuclear antibodies (ANA)^	^Negative (Negative)^
^Anti-Sjögren's syndrome-related antigen A (SSA)^	^Negative (Negative)^
^Anti-Sjögren's syndrome-related antigen B (SSB)^	^Negative (Negative)^
^Anti-double-stranded DNA antibodies (DNA)^	^Negative (Negative)^
^Beta-2 glycoprotein (B2GP),^	^Negative (Negative)^
^Lupus anticoagulant (LAC)^	^Negative (Negative)^
^Anti-myeloperoxidase (MPO)^	^Negative (Negative)^
^Anti PR3^	^Negative (Negative)^
^Erythrocyte sedimentation rate (ESR)^	^99 mm/hr ^ ^(0-20 mm/hr)^
^C-reactive protein^	^2.4 mg/L^ ^ (<1.0 mg/L)^

The patient was sent for a right submandibular gland core biopsy, which showed salivary gland tissue with dense nodular lymphocytic infiltrate, variable parenchymal fibrosis, and salivary gland acinar atrophy, reported as consistent with SS. Specialty pathology review was requested by the clinical team due to clinical suspicion of IgG4-RD, due to high serum IgG4+ cells on IgG subclass analysis (Table 2). Further pathology review showed lymphoplasmacytic infiltrate, fibrosis, and increased IgG4-positive plasma cells, greater than a hundred/high-power field in some areas, representing approximately 50% of plasma cells, thus supporting the diagnosis of IgG4-RD.

**Table 2:** Serologic laboratory results of IgG total and subclasses on

^Serologic marker^	^Pre-treatment values (Reference range)^	^Post-treatment values (Reference range)^
^IgG subclasses (1-4)^	^3535 mg/dL (586-1602 mg/dL)^	^683 mg/dL (586-1602 mg/dL)^
^IgG subclass 1^	^1439 mg/dL (248-810 mg/dL)^	^335 mg/dL (248-810 mg/dL)^
^IgG subclass 2^	^277 mg/dL (130-555 mg/dL)^	^114 mg/dL (130-555 mg/dL)^
^IgG subclass 3^	^>216 (15-102 mg/dL)^	^48 mg/dL^ ^(15-102 mg/dL)^
^IgG subclass 4^	^843 mg/dL (2-96 mg/dL)^	^89 mg/dL (2-96 mg/dL)^

**Figure 2:**
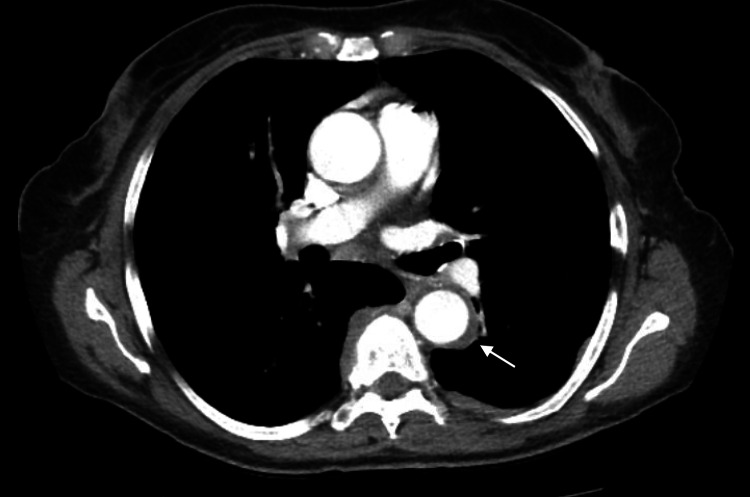
Bilateral mediastinal lymphadenopathy with 6 mm thickened pleura surrounding the descending aorta (arrow)

Further workup was done to rule out occult malignancy. CT with contrast showed nodular pleural thickening bilaterally, more prominent in the left lobe with 6 mm thickened pleura surrounding the mid-descending thoracic aorta (Figure 2), small nodules in the left lower lobe, mildly enlarged bilateral hilar and mediastinal lymphadenopathy, a new 4 cm long right paraspinal soft tissue mass at T6-8, abnormal enlargement and edematous changes of bilateral kidneys, mildly enlarged retroperitoneal nodes as well as bilateral pelvic sidewall, common femoral, and left inguinal lymphadenopathy.

PET/CT showed intense diffuse increased hypermetabolic activity in the bilateral submandibular, sublingual, and parotid glands. There was increased hypermetabolic activity in pleural nodularity in both lungs, lymph nodes in the neck, chest, abdomen, and pelvis, and the renal parenchyma. The highest uptake ranged from 7.7 to 9.8 in the submandibular glands, which were enlarged on the CT images and increased in size since the previous CT (one year prior). There was no abnormal hypermetabolic activity to suggest malignancy.

Upon further workup, the patient was noted to have increased M protein (1.26 g/dl) on protein electrophoresis of the serum (SPEP). Bone marrow aspirate showed rare plasma cells with a clonal IgG kappa plasma cell population, 2% of the total. Urine protein electrophoresis (UPEP) was negative. The free light chain (FLC) ratio was 1.61. The patient did not have any lytic bone lesions or hypercalcemia; thus, a diagnosis of monoclonal gammopathy of undetermined significance (MGUS) with a 2% clonal population was made. Follow-up SPEP, which was four months after treatment for IgG4-RD, showed resolution of this monoclonal gammopathy with an unremarkable SPEP and normal immunofixation without any evidence of monoclonal proteins.

**Figure 3:**
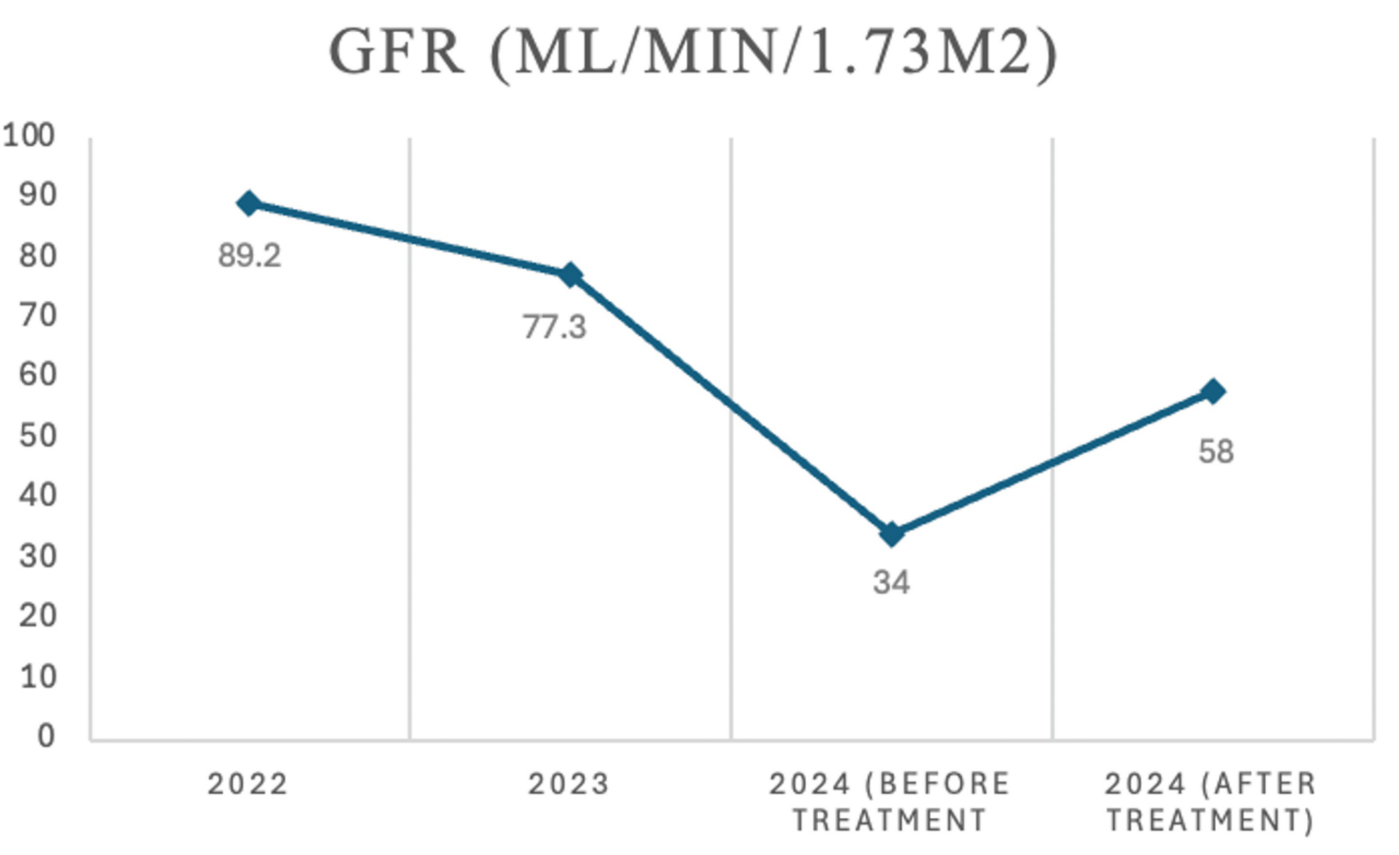
Estimated glomerular filtration rate (eGFR) changes

**Figure 4:**
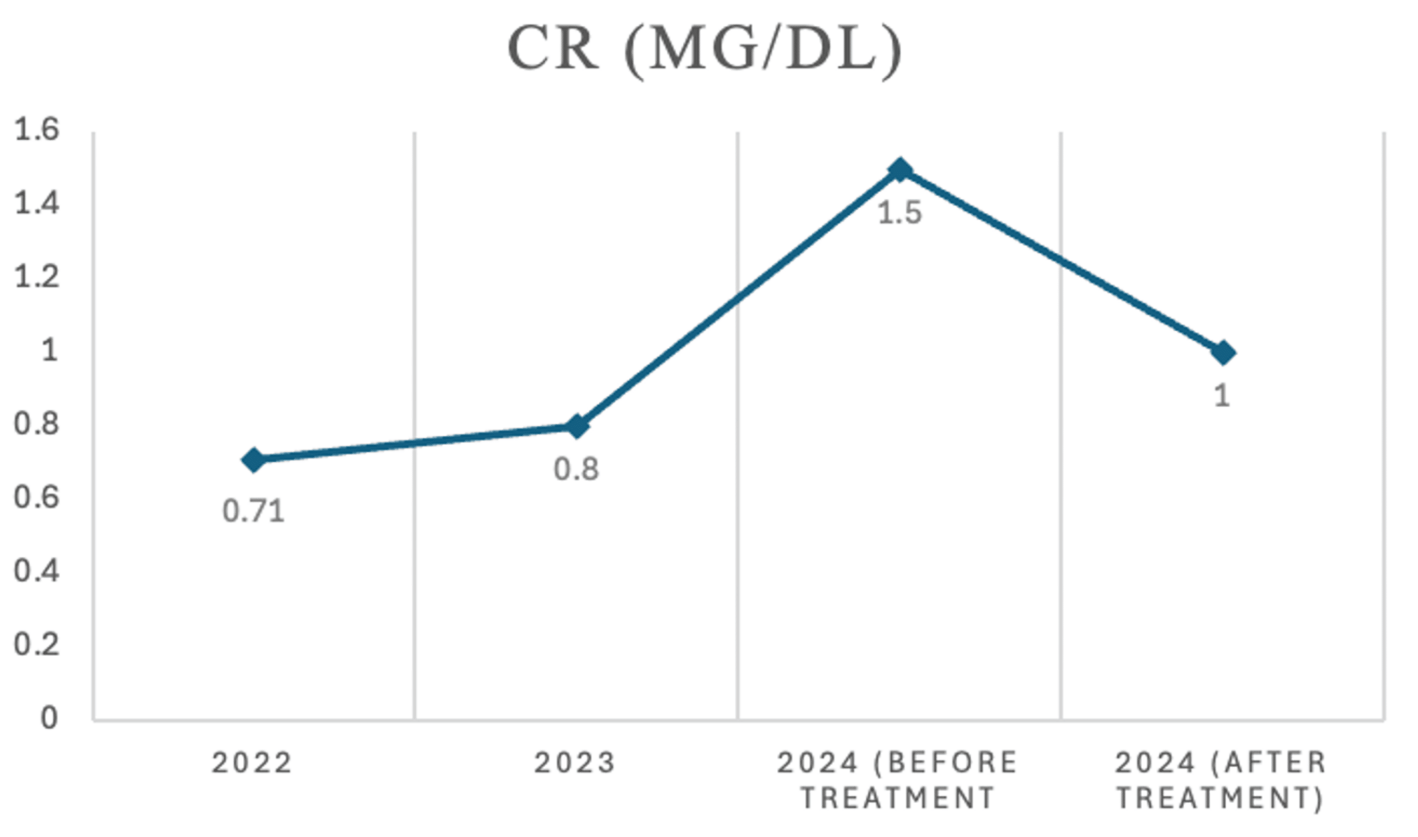
Creatinine changes

Systemic involvement of IgG4-RD was noted, including nodular pleural thickening with clinical dyspnea on exertion and pulmonary function testing (PFT), which showed obstructive defect with air trapping and decreased oxygen diffusion (DLCO) (Table 3). The patient also had retroperitoneal fibrosis with encasement of the aorta and kidney infiltration with resultant decline in the eGFR (Figures 3, 4).

**Table 3:** Pulmonary function test FVC: functional vital capacity; FEV1: forced expiratory volume; TLC: total lung capacity; DLCO: decreased oxygen diffusion

^Parameter^	^Result^
^FVC^	^1.78L (81%)^
^FEV1^	^1.29L (76%)^
^TLC^	^4.6L (97%)^
^DLCO^	^54%^

**Figure 5:**
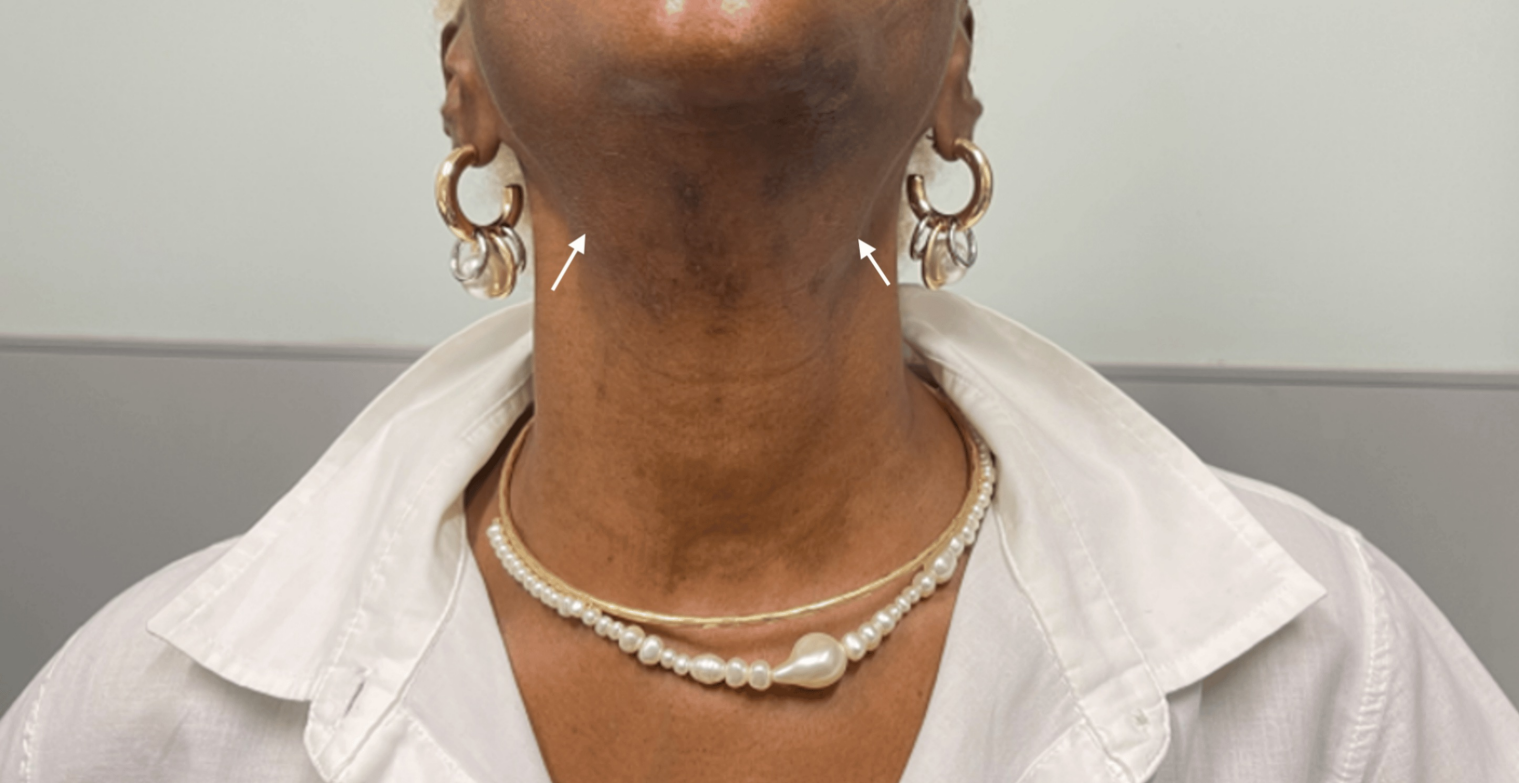
After treatment with prednisone and rituximab The submandibular glands regressed completely and were no longer palpable (arrow)

A clinical diagnosis of IgG4-RD was made, and the patient was started on prednisone 60 mg a day. At her follow-up visit a month later, the patient reported significant improvement in symptoms, including a return of appetite, significant improvement in her mouth dryness, no further dyspnea on exertion, and a reduction in the size of her submandibular glands. The patient was then treated with rituximab 1000 mg x two doses and was started on Bactrim 400-80 MG for infection prophylaxis. During the course of her induction treatment with high-dose steroids and rituximab, the patient experienced two infectious complications, a submental abscess and a fingertip felon, both of which resolved with antibiotic care; however, they delayed her second rituximab infusion. Within several months, the submandibular glands regressed completely and were no longer palpable (Figure 5). Repeat laboratory testing showed significant improvement in her IgG4 levels, etc. (Table 2). Prednisone was successfully tapered, and the patient remained symptom-free at the time of this writing, nearly six months after the diagnosis.

## Discussion

IgG4-RD can manifest in any organ, and clinical symptoms are variable. It may be confused with malignancy, infection, or autoimmune diseases such as SS or vasculitis [5]. In our patient, the diagnosis was further confused by the findings of sicca syndrome and positive autoantibodies for RF and CCP. Diagnosis ultimately required a core biopsy of the salivary gland and specialty pathology review. Upon deeper multisystem workup, the patient was found to have occult lung involvement, retroperitoneal fibrosis with encasement of the aorta, and kidney infiltration. It is noteworthy that this case took two years for appropriate specialty referral and diagnosis. Unfortunately, during this time, the disease progressed with multisystem disease, as evidenced by new findings on repeat CT scans and with the decline of kidney function.

The diagnosis of IgG4-RD is very challenging, as clinical manifestations overlap with other conditions and mimic a variety of neoplastic, inflammatory, and infectious conditions [7]. As such, most patients experience a delay in the diagnosis of IgG4-RD. A single-center retrospective study by Pucar et al. (2019) showed considerable delay in the diagnosis of IgG4-RD, with the median time to diagnosis of 64 months [11]. According to the study by Dassanayaka W et al. (2023), even patients with suspicion for IgG4-RD and tissue biopsy with staining for such were challenging to classify using the histologic diagnosis of IgG4-RD. Among this cohort of 204 cases, 54 cases had typical storiform fibrosis and 65 cases had >10 IgG4+ plasma cells [12]. On final histopathologic review, only 14/204 cases (6.78%) were ultimately classified as highly suggestive of IgG4-RD [12].

We performed an extensive diagnostic workup to exclude malignancy in our patient. Multiple studies show increased overall risk of oncologic diseases in patients with IgG4-RD with a standardized incidence ratio (SIR) of 2.57 [13,14,15]. Specifically, the risk of pancreatic cancer (SIR 4.07) and lymphoma (SIR 69.17) is particularly high [13]. There is also evidence that malignancy can sometimes predispose patients to develop IgG4-RD [16]. Differentiating the diagnosis between IgG4-RD and cancer is particularly challenging, especially when distinguishing it from lung or hematologic cancers, as IgG4-RD can manifest as a solitary parenchymal mass or lung nodules [17]. Multiple studies show the utility of PET/CT for IgG4-RD, especially the 18F-fluorodeoxyglucose (18F-FDG) PET/CT modality [18]. 18F-FDG PET/CT has a high sensitivity of 85.7% and moderate specificity of 66.1% for diagnosing IgG4-RD [19]. The presentation of the patient in our case was concerning for malignancy, with notable features including significant weight loss, prominent mediastinal and hilar lymphadenopathy, abnormal nodular pleural thickening, and a sclerotic bone lesion of T7. A PET/CT scan in our patient effectively ruled out malignancy, as there was no hypermetabolic activity. Furthermore, the patient’s rapid response to corticosteroid therapy was reassuring and retrospectively strengthened the evidence of IgG4-RD diagnosis.

Our patient in our case also had an M spike on SPEP and immunofixation electrophoresis (IFE), suggesting MGUS. There is limited research on the relationship between IgG4-RD and MGUS. Some studies suggest that the high levels of a single IgG4 isotype in IgG4-RD can resemble MGUS, potentially leading to its misinterpretation as a monoclonal gammopathy [20]. A high concentration of polyclonal IgG4 can create an M spike, potentially resembling a monoclonal band on SPEP, and is more commonly observed in patients with renal impairment, such as our patient [21]. However, in our patient, monoclonality was confirmed by bone marrow aspiration, which confirmed a clonal IgG kappa plasma cell population, and the patient met all criteria for MGUS. Notably, follow-up SPEP and IFE after initiation of IgG4-RD treatment did not reveal any evidence of MGUS. The literature on the phenomenon of MGUS resolution is limited, highlighting the need for further investigation into the relationship between MGUS and IgG4-RD.

IgG4-RD has an excellent response to prednisone, to the extent that response to prednisone is listed as a diagnostic criterion for diagnosing IgG4-RD [10]. Response usually occurs within two to four weeks, which is in keeping with the prompt response observed in our patient [20]. Rituximab is described to improve remission rates and lower the rate of relapse [22]. Due to the multisystem nature of our patient’s disease, rituximab was started in combination with steroids for induction of remission. This case also highlights the potential toxicities of such immunosuppressive therapy, as our patient sustained two new infections during the course of her induction therapy, a submental abscess and a felon, both of which were successfully treated with oral antibiotics but which delayed her second rituximab infusion.

## Conclusions

IgG4-RD is challenging to diagnose and needs to be considered when atypical forms of autoimmune-type disease are encountered. It needs to be carefully distinguished from SS and malignancy. It is crucial to consider IgG4-RD and to do a multisystem workup to assess organ damage and tailor therapies accordingly. In this case, the diagnosis was guided by a high serum IgG4 level; however, IgG4 is not always elevated and can be high in some patients without IgG4-RD; thus, biopsy confirmation with special stains and specialty review is essential. Prompt recognition and management of IgG4-RD can improve patient outcomes and prevent long-term morbidity.
